# P-1171. C. the Difference: A Daily Review of *Clostridioides difficile* Order Appropriateness in a Pediatric Acute Care Hospital

**DOI:** 10.1093/ofid/ofae631.1357

**Published:** 2025-01-29

**Authors:** Dara M Czernikowski, Sharon Karunakaran, Margot Stein, Jillian Regier, Christina Jockel, Rhianna Kozinski, Hannah M Creager, Michael D Green, Lindsay Montoya

**Affiliations:** UPMC Children's Hospital of Pittsburgh, Pittsburgh, Pennsylvania; UPMC Children's Hospital of Pittsburgh, Pittsburgh, Pennsylvania; UPMC Children's Hospital of Pittsburgh, Pittsburgh, Pennsylvania; UPMC Children’s Hospital of Pittsburgh, Pittsburgh, Pennsylvania; UPMC Children's Hospital of Pittsburgh, Pittsburgh, Pennsylvania; UPMC Children's Hospital of Pittsburgh, Pittsburgh, Pennsylvania; University of Pittsburgh Medical Center, Pittsburgh, Pennsylvania; University of Pittsburgh School of Medicine, Pittsburgh, PA; UPMC Children's Hospital of Pittsburgh, Pittsburgh, Pennsylvania

## Abstract

**Background:**

*Clostridioides difficile* (CD) infections (I) are prevalent and costly healthcare-associated infections (HAIs). High rates of CD colonization in younger children make differentiation between colonization and infection challenging. Assessing for presence of CDI risk factors and absence of alternative causes of diarrhea can help to optimize diagnosis and avoid unnecessary CD testing.

**Goal**: Optimize HA CD testing and reduce HA CDIs at a pediatric acute care hospital.

**Figure:**
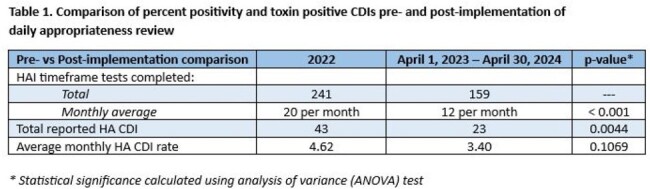

**Methods:**

Beginning mid-March 2023, CD tests were shifted to batch testing once daily to allow for pre-review of pending HAI timeframe (collected on or after the 3rd calendar day of admission) test orders. Orders were first identified through a prompted report and associated charts were reviewed using standardized criteria to determine appropriateness: presence of risk factors and relevant symptoms in the absence of other explanations for diarrhea (laxatives, enemas, underlying conditions, etc.). Discussions with clinical teams were initiated when testing was deemed inappropriate. Orders were canceled if teams agreed with reviewer’s feedback. Outcome and process data were retrospectively compared with one year of pre-implementation data. The Health System QI Committee approved this project.

**Figure:**
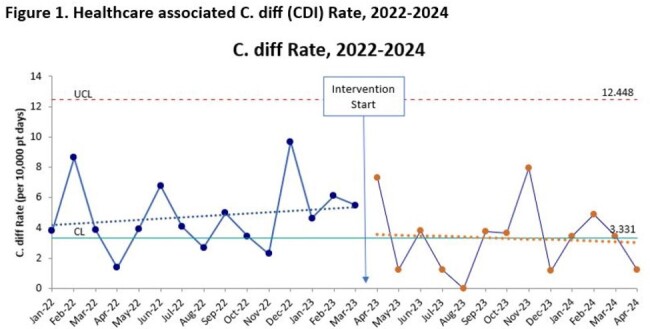

**Results:**

From April 1, 2023 – April 30, 2024, 216 pending HAI timeframe CD tests were reviewed: 83 (39%) were deemed inappropriate; 60 (72%) were canceled by the ordering physicians. Of the total 159 HAI timeframe orders run, 23 (19%) resulted positive. The number of completed HAI timeframe CD tests, average monthly HA CDI rate, and reported HA CDIs all decreased post-implementation (Table 1). The observed HA CDI rate also decreased post-implementation (Fig 1). Using the number of canceled orders and 18% HA CDI positivity rate observed in 2022, we estimate avoidance of 11 misdiagnosed CDIs in CD colonized children from this initiative.

**Conclusion:**

Implementation of the daily review protocol decreased the number of HAI tests performed and the HA CDI rate. Next steps to advance this work include implementing electronic initiatives such as blocking CD tests with recent laxative use, and pop-up reminders for providers to consider testing patients admitted with CD symptoms & risk factors.

**Disclosures:**

**Michael D. Green, MD, MPH**, ADMA: Advisor/Consultant|Bristol Myers Squibb: Advisor/Consultant|ITB-MED: Advisor/Consultant|kamada: Honoraria

